# Supplementary material for: Taming active turbulence with patterned soft interfaces
Source: Nat Commun. 2017 Sep 15;8:564. doi: 10.1038/s41467-017-00617-1 (PMC5601458; doi:10.1038/s41467-017-00617-1)
Supplement: Supplementary file 2 — Supplementary Information [file 41467_2017_617_MOESM2_ESM.pdf]

Description of Supplementary Files

File Name: Supplementary Information

Description: Supplementary figures

File Name: Supplementary Movie 1

Description: Turbulent active nematic. Fluorescence micrographs of the active nematic in contact with an isotropic oil.

File Name: Supplementary Movie 2

Description: Active nematic swirls in contact with toroidal focal conic domains (TFCDs).

File Name: Supplementary Movie 3

Description: Influence of the activity on the defect trajectories of confined active nematics.

File Name: Supplementary Movie 4

Description: Preservation of the topological charge inside active swirls.

File Name: Supplementary Movie 5

Description: Active nematic swirls are metastable.

File Name: Supplementary Movie 6

Description: Active nematic swirls induce localized flows.

File Name: Peer Review File

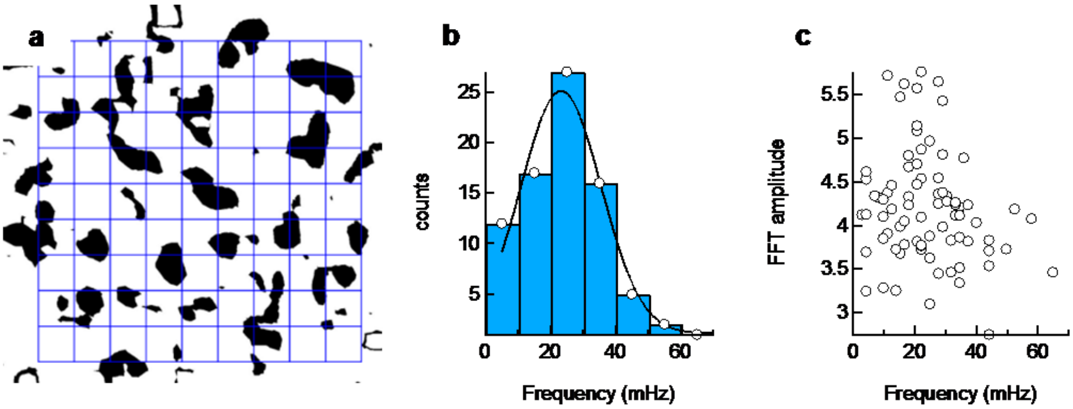

Supplementary Figure 1. Characteristic frequency of the active nematic in the turbulent regime. (**a**) Snapshot of the AN in the unconstrained turbulent regime for the same conditions described in Fig. 1 of the main text. Here, the fluorescence images have been binarized considering the Okubo-Weiss parameter and the criterion $\mathrm{OW} = (\partial_x v_x)^2 + \partial_y v_x \cdot \partial_x v_y < 0$. To obtain a statistical assessment of the vortex lifetime in this regime, we divide the area in 30x30 μm^2 cells, a size consistent with the characteristic length scale extracted from the distribution of vortex sizes (see main text and Fig. 1). We then measure the area fraction with OW < *0* for each of the cells as a function of time (during 1000 s), and we compute the Fast Fourier Transform (FFT) of this signal. (**b**) Distribution of characteristic frequencies for the ensemble of 81 boxes in **a**. The solid line is a Gaussian fit. (**c**) Height of the highest peak in the power spectra of the data in **a** as a function of its frequency, for the ensemble of 81 boxes.

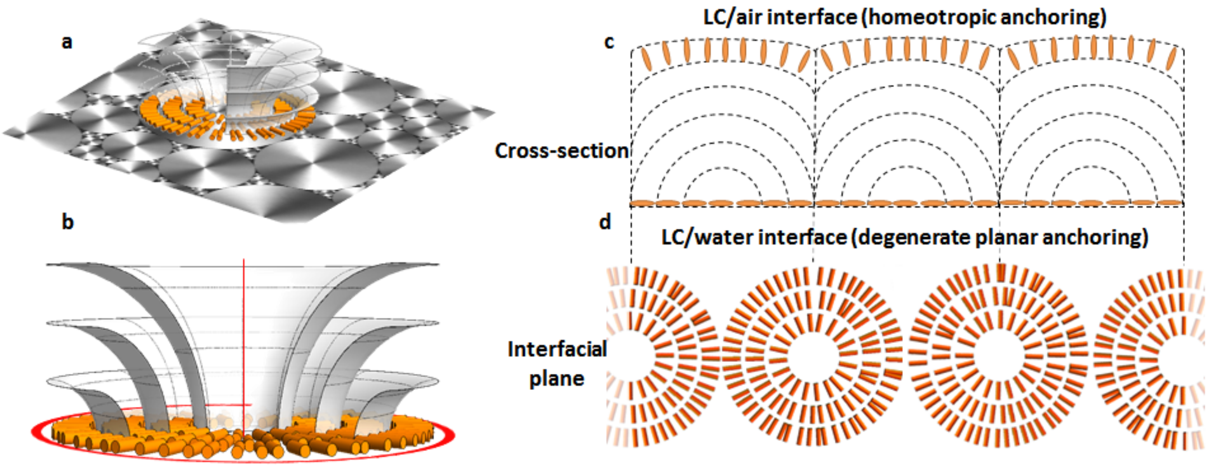

Supplementary Figure 2: Structure of the TFCDs. a Confocal reflection micrograph (field of view 300 x 300 μm^2) showing the toroidal focal conic domains at the 8CB/water interface. A sketch of the 3D SmA planes and the orientation of the mesogen molecules on top of a domain is included. **b** SmA lamellae bend around two singular lines (depicted in red in the sketch) namely, a circle at the LC/water interface, and a straight line running from the circle center into the bulk of the LC. **c** Cross section of the bulk structure of the lamellar planes in the LC across the sample thickness. **d** Organization of the LC molecules at the interface.

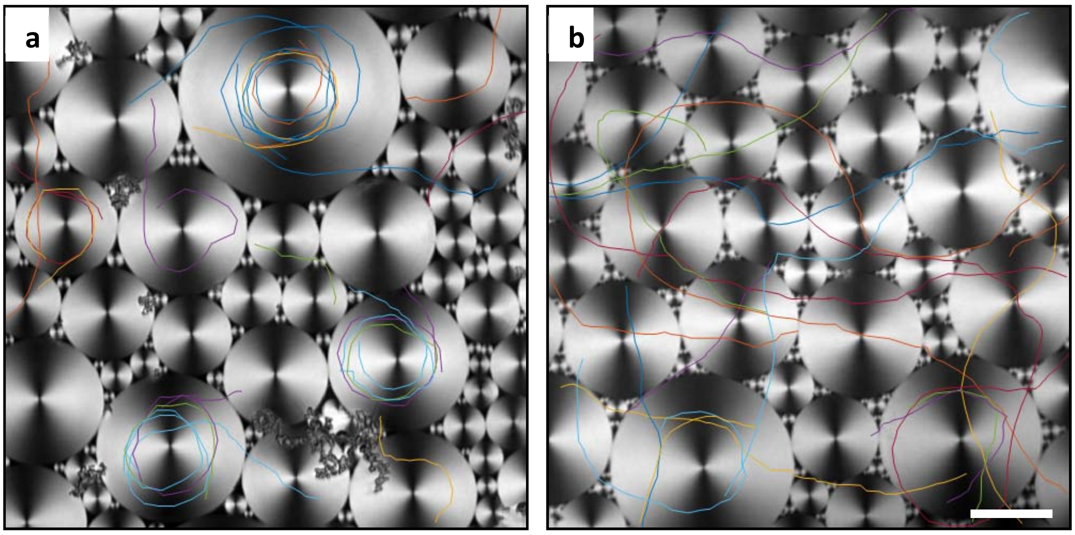

Supplementary Figure 3. Trapping of defects by Toroidal Focal Conic Domains. **a** Confocal reflection micrograph of different TFCD and overlaid trajectories of +1/2 defects. Each color corresponds to a different defect. Experimental conditions of the AN are [ATP] = 700 μM, [PEG] = 0.8%w/w. b Similar study but with experimental conditions of the AN are [ATP] = 70 μM, [PEG] = 0.8%w/w. The scale bar is 25 μm.

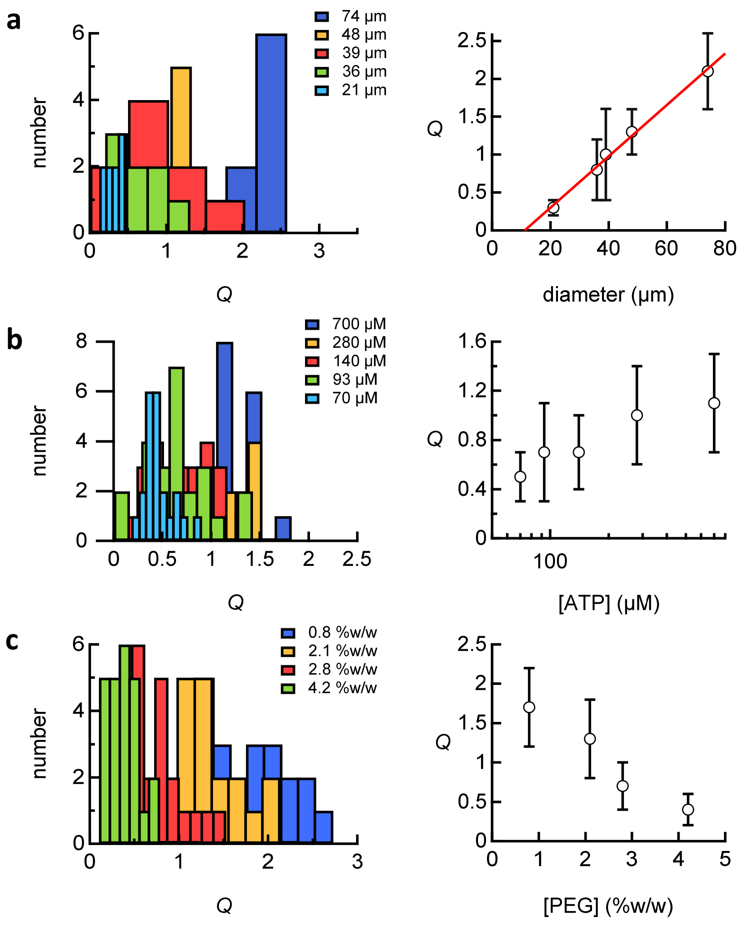

Supplementary Figure 4. Distribution of winding number as a function of different control parameters. a Dependence of *Q* with TFCD diameter, for experiments performed with [ATP] = 700 μM and [PEG]=0.8 %w/w. The line is a linear fit to the data. **b** Dependence of *Q* with [ATP] for circulation around TFCD with diameter 60 μm, and experiments performed with [PEG] = 1.6 %w/w. **c** Dependence of Q with [PEG] for circulation around TFCD with diameter 60 μm, and experiments performed with [ATP] = 1.4 mM. Error bars are the standard deviation for the measurements under each condition.

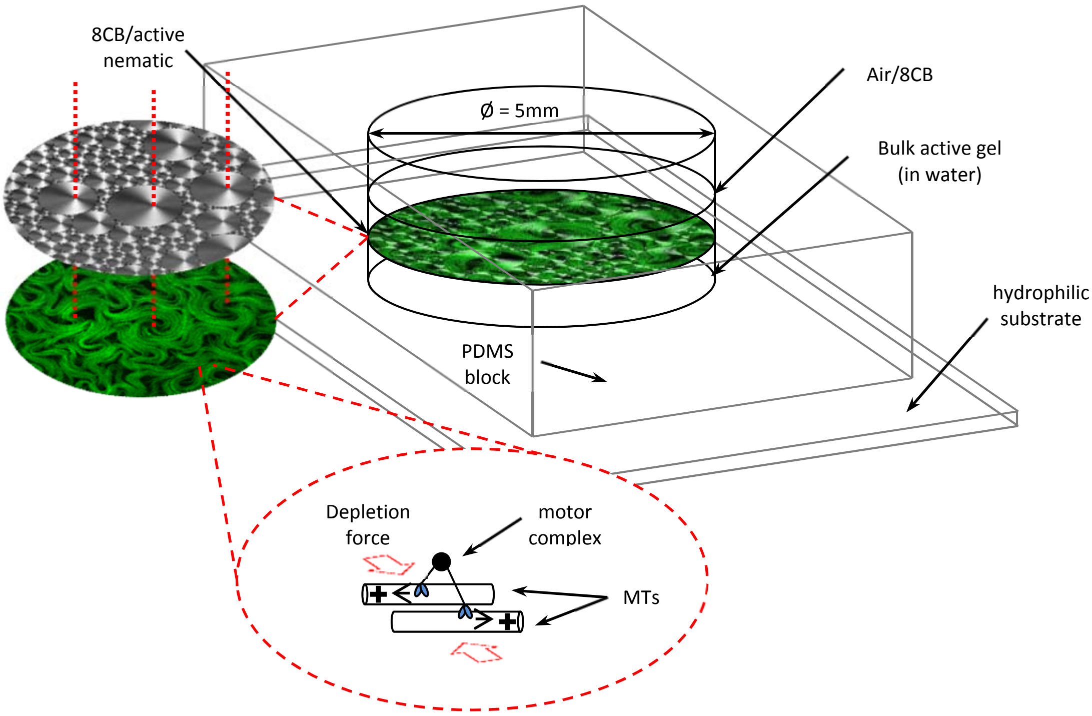

Supplementary Figure 5: Sketch of the experimental setup. The active nematic forms at the LC/water interface contained in the open cavity of a custom PDMS block, where all the experiments are performed.
